# Supplementary material for: Opportunities for improved HIV prevention and treatment through budget optimization in Eswatini
Source: PLoS One. 2020 Jul 23;15(7):e0235664. doi: 10.1371/journal.pone.0235664 (PMC7377429; doi:10.1371/journal.pone.0235664)
Supplement: S2 Fig — (DOCX) [file pone.0235664.s002.docx]

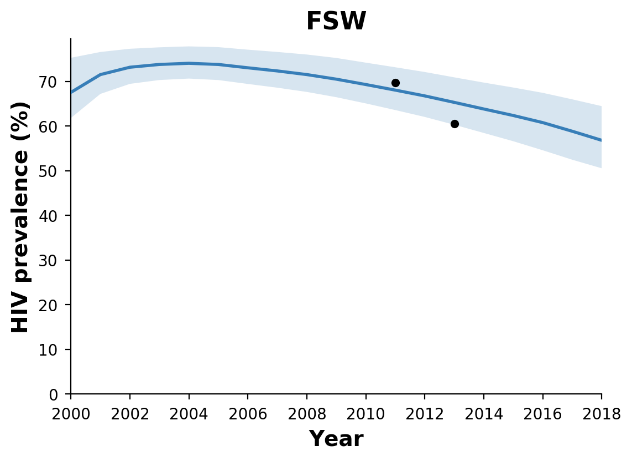

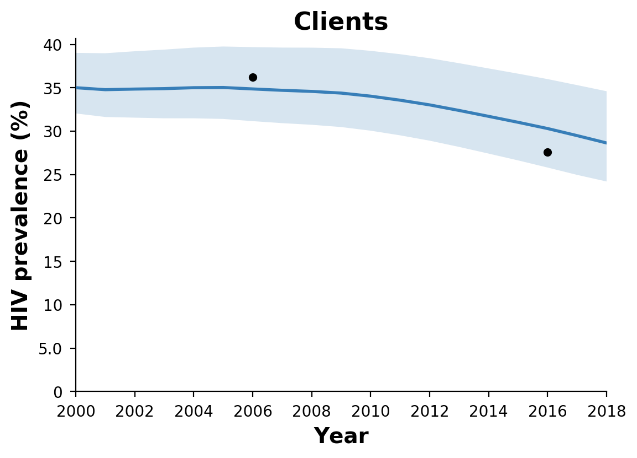


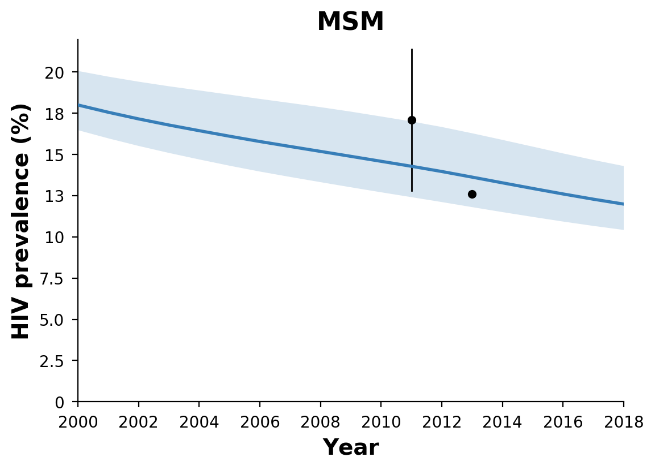


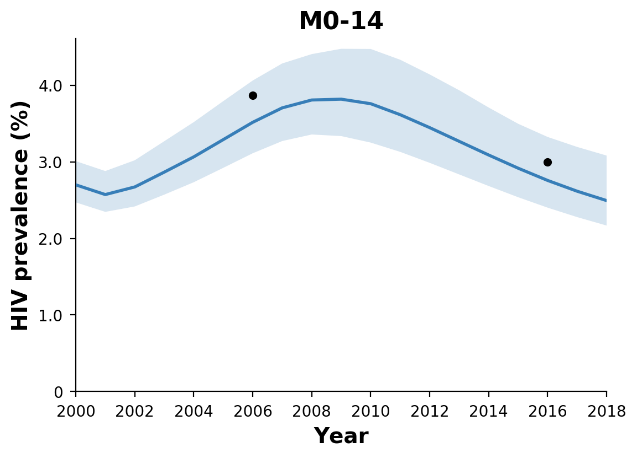

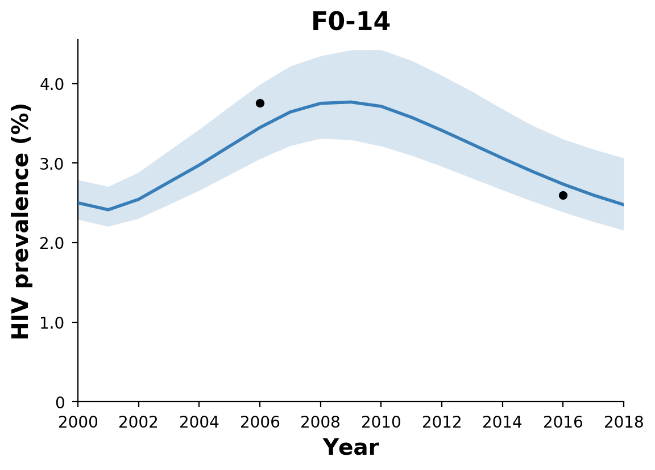


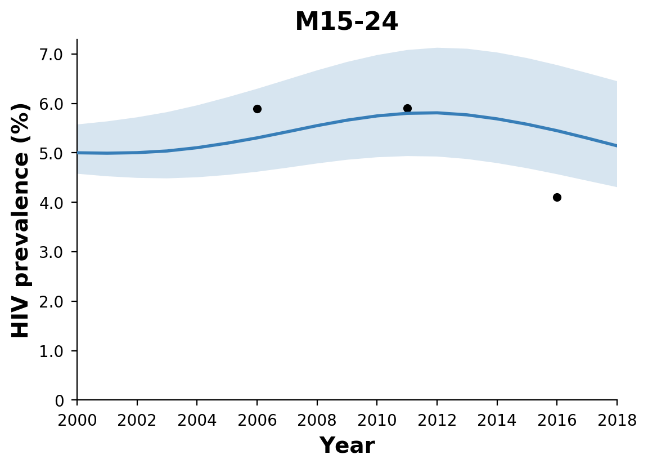


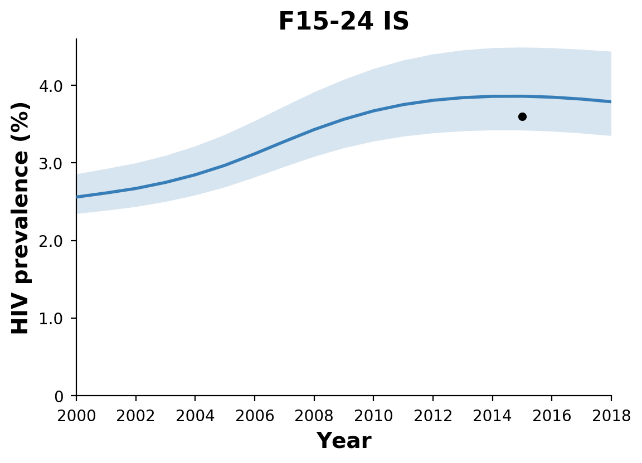

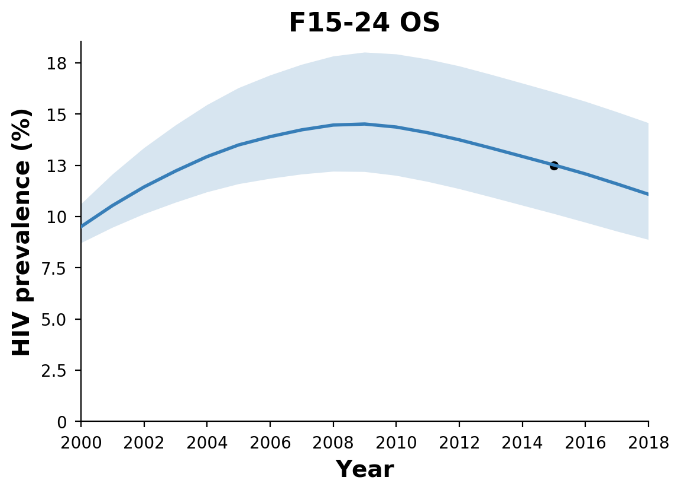


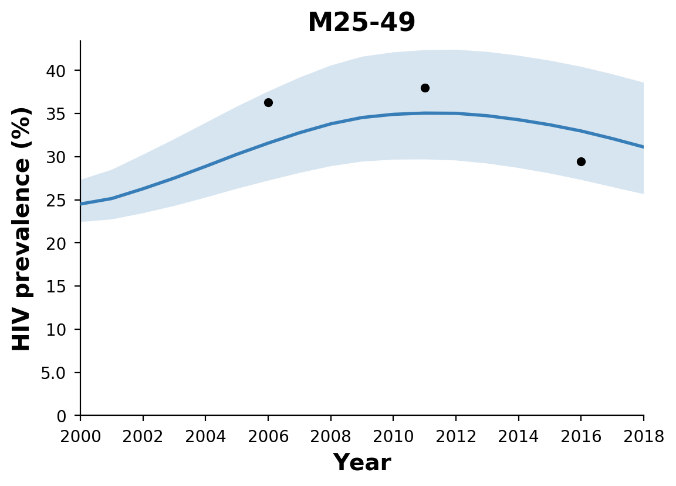

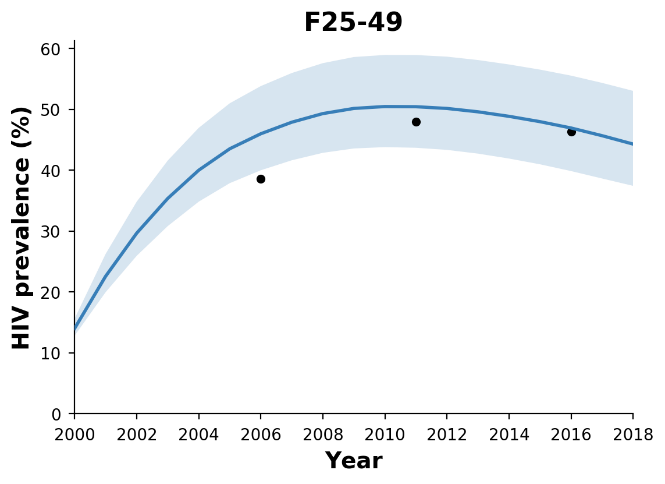


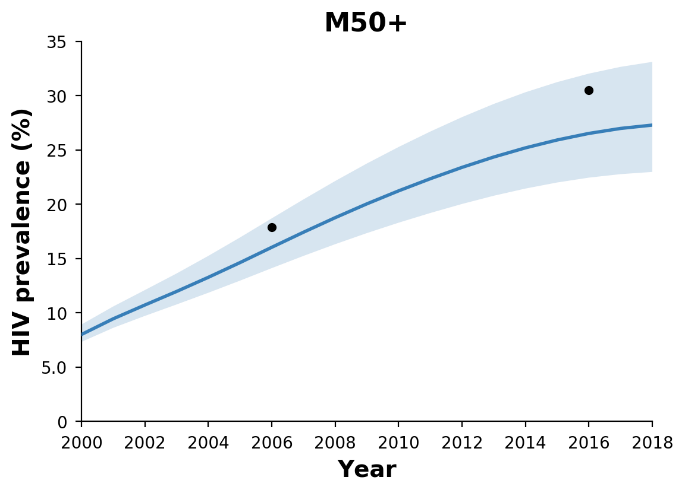

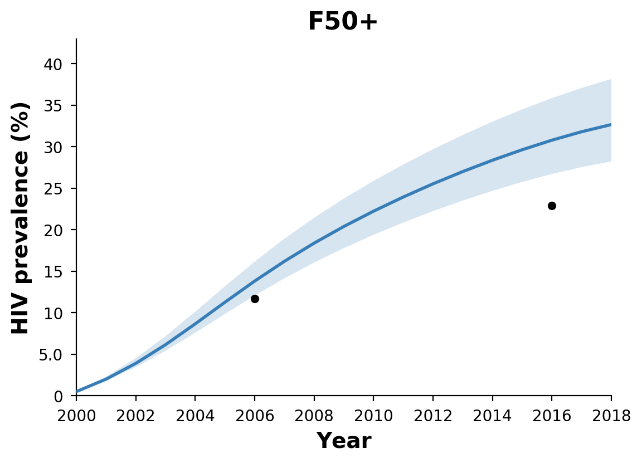


Figure S2. Model calibration to HIV prevalence estimates by population

IS = in school. OS = out-of-school.

Source: Optima HIV model, 2018
