# Supplementary material for: Opportunities for improved HIV prevention and treatment through budget optimization in Eswatini
Source: PLoS One. 2020 Jul 23;15(7):e0235664. doi: 10.1371/journal.pone.0235664 (PMC7377429; doi:10.1371/journal.pone.0235664)
Supplement: S3 Fig — (DOCX) [file pone.0235664.s003.docx]

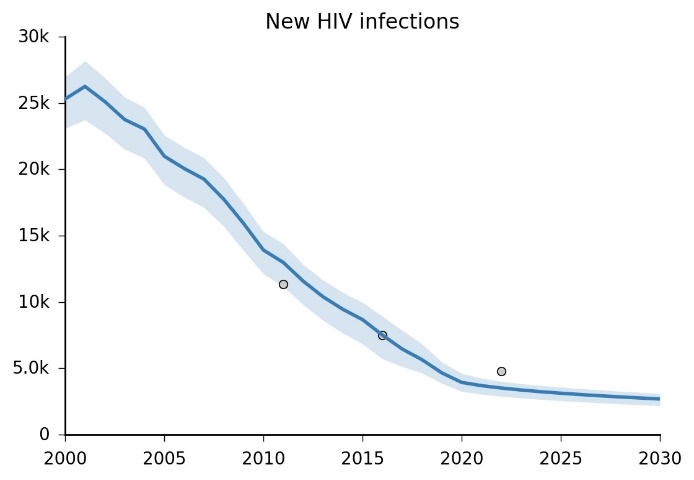

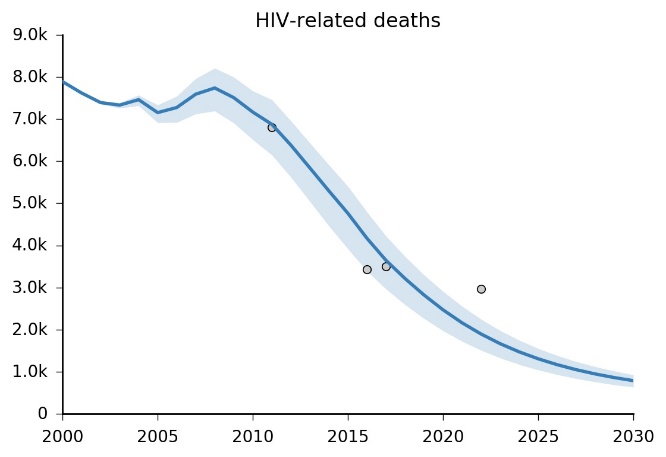


**Year**

**Year**

**HIV-related deaths**

**New HIV infections**

Figure S3. Model calibration to new HIV infections and HIV-related deaths with projections to 2030 with uncertainty bound shading

Source: Optima HIV model, 2018
