# Supplementary material for: Opportunities for improved HIV prevention and treatment through budget optimization in Eswatini
Source: PLoS One. 2020 Jul 23;15(7):e0235664. doi: 10.1371/journal.pone.0235664 (PMC7377429; doi:10.1371/journal.pone.0235664)
Supplement: S1 Table — (DOCX) [file pone.0235664.s004.docx]

Table S1. HIV prevalence estimates

| **Population** | **HIV prevalence** | **Year HIV prevalence**  **last reported** | **Population size for year HIV prevalence last reported** ^e^ | **Estimated PLHIV** | **Proportion of total PLHIV** |
| --- | --- | --- | --- | --- | --- |
| Female sex workers (FSW) | 60.5%^a^ | 2013 | 3,930 | 2,377 | 1.2% |
| Clients of FSW | 27.6%^b^ | 2016 | 12,296 | 3,394 | 1.8% |
| Men who have sex with men (MSM) | 12.6%^c^ | 2013 | 2,341 | 295 | 0.2% |
| Females 0-14 years | 2.6%^b^ | 2016 | 192,925 | 5,016 | 2.6% |
| Males 0-14 years | 3.0%^b^ | 2016 | 193,928 | 5,818 | 3.1% |
| Females 15-24 years in-school | 3.6%^d^ | 2015 | 57,769 | 2,080 | 1.1% |
| Females 15-24 years out-of-school | 12.5%^d^ | 2015 | 53,055 | 6,627 | 3.5% |
| Males 15-24 years | 4.1%^b^ | 2016 | 107,187 | 4,395 | 2.3% |
| Females 25-49 years | 46.3%^b^ | 2016 | 172,432 | 79,921 | 42.0% |
| Males 25-49 years | 29.4%^b^ | 2016 | 156,910 | 46,163 | 24.2% |
| Females 50 years and older | 22.9%^b^ | 2016 | 77,262 | 17,688 | 9.3% |
| Males 50 years and older | 30.5%^b^ | 2016 | 54,813 | 16,734 | 8.8% |

PLHIV = people living with HIV

^a^USAID. HIV among female sex workers and men who have sex with men in Swaziland: A combined report of quantitative and qualitative studies. Research to Prevention (R2P), Johns Hopkins Center for Global Health, Baltimore, MD, USA; 2013

^b^PEPFAR. Swaziland HIV Incidence Measurement Survey (SHIMS) 2: Summary sheet, preliminary findings 2016-2017. Washington, DC, USA; 2017

^c^Baral S, Grosso A, Mnisi Z, Adams D, Fielding-Miller R, Mabuza X, et al. Examining prevalence of HIV infection and risk factors among female sex workers (FSW) and men who have sex with men (MSM) in Swaziland. Research to Prevention (R2P), Johns Hopkins Center for Global Health, Baltimore, MD, USA; 2013

^d^Sitakhela Likusasa Impact Evaluation Project - IHM Southern Africa. Baseline value. 2018

^e^United Nations World Population Prospects; Geneva, Switzerland; 2017. Available from: <https://population.un.org/wpp/>
