# Supplementary material for: Opportunities for improved HIV prevention and treatment through budget optimization in Eswatini
Source: PLoS One. 2020 Jul 23;15(7):e0235664. doi: 10.1371/journal.pone.0235664 (PMC7377429; doi:10.1371/journal.pone.0235664)
Supplement: S2 Table — (DOCX) [file pone.0235664.s005.docx]

Table S2. HIV testing modality coverage, yield, and mean saturation

| **HIV testing modality** | **Coverage** | **Yield** | **Target population** | **Mean saturation** | **Year** |
| --- | --- | --- | --- | --- | --- |
| HIV testing - home-based | 418 | 1.3% | Total population | 50% | 2017 |
| HIV testing - index | 2,950 | 57.8% | Total population | 10% | 2017 |
| HIV testing - mobile | 65,087 | 4.5% | Adults 15+ years | 60% | 2017 |
| HIV testing - other PITC | 229,202 | 6.4% | Total population | 90% | 2016 |
| HIV testing - self-testing | 2,242 | 3.6% | Adults 15+ years | 30% | 2017 |
| HIV testing - VCT | 76,056 | 11.4% | Adults 15+ years | 30% | 2016 |

PITC = provider-initiated testing and counselling

VCT = voluntary testing and counselling
