# Supplementary material for: Opportunities for improved HIV prevention and treatment through budget optimization in Eswatini
Source: PLoS One. 2020 Jul 23;15(7):e0235664. doi: 10.1371/journal.pone.0235664 (PMC7377429; doi:10.1371/journal.pone.0235664)
Supplement: S3 Table — (DOCX) [file pone.0235664.s006.docx]

Table S3. ARV refill modality coverage, efficacy, and saturation

| **ART refill modality** | **Coverage** | **Efficacy** | **Saturation** | | **Year** |
| --- | --- | --- | --- | --- | --- |
|  |  |  | **Low** | **High** |  |
| Community-based group ART | 660 | 81% retention (19% LTFU) | 20% | 45% | 2016 |
| Facility-based group ART | 2,133 | 96% retention (4% LTFU) | 30% | 50% | 2016 |
| Fast-Track ART | 3,514 | 99% retention (1% LTFU) | 50% | 60% | 2016 |
| Mainstream ART | 139,953 | 55% retention (45% LTFU) | 100% | 100% | 2016 |
| Outreach ART | 1,014 | 77% retention (23% LTFU) | 70% | 80% | 2016 |

LTFU = loss to follow-up
