# Supplementary material for: Opportunities for improved HIV prevention and treatment through budget optimization in Eswatini
Source: PLoS One. 2020 Jul 23;15(7):e0235664. doi: 10.1371/journal.pone.0235664 (PMC7377429; doi:10.1371/journal.pone.0235664)
Supplement: S4 Table — (DOCX) [file pone.0235664.s007.docx]

Table S4. ART refill modality coverage constraints and saturation ranges

| **ART refill modality** | **Coverage constraint*** | | **Saturation range** |
| --- | --- | --- | --- |
|  | **Minimum** | **Maximum** |  |
| Community-based group ART | 0 | 32,900 | 20%-45% |
| Facility-based group ART | 0 | 49,400 | 30%-50% |
| Fast-Track ART | 0 | 82,350 | 50%-60% |
| Mainstream ART | 55,000 | None | 100%-100% |
| Outreach ART | 1,000 | 115,300 | 70%-80% |

*Constraints are based on eligibility for being switched to alternate ARV refill modalities and/or to represent treatment retention rates

Full model parameters details are available from [optimamodel.com/parameter-data-sources](file:///G:\My%20Drive\Optima%20research\Optima%20HIV%20research\WIP\17.%20eSwatini\Draft%20paper\7.%20To%20be%20submitted_20190715\optimamodel.com\parameter-data-sources).
