# Supplementary material for: Opportunities for improved HIV prevention and treatment through budget optimization in Eswatini
Source: PLoS One. 2020 Jul 23;15(7):e0235664. doi: 10.1371/journal.pone.0235664 (PMC7377429; doi:10.1371/journal.pone.0235664)
Supplement: S5 Table — (DOCX) [file pone.0235664.s008.docx]

Table S5. Baseline and optimised HIV budget allocations and coverage

| **HIV programs** | **Baseline**  **budget** | **Optimised**  **budget** | **% change**  **in budget** | **Baseline**  **coverage** | **Optimised**  **coverage** | **% change**  **in coverage** |
| --- | --- | --- | --- | --- | --- | --- |
| Antiretroviral therapy (ART) | $39,521,381 | $39,521,381 | 0% | 174,107 | 174,107 | 0% |
| HIV testing services (HTS) | $4,190,502 | $7,295,083 | 74% | 347,654 | 536,410 | 54% |
| Prevention of mother-to-child transmission (PMTCT) | $7,232,881 | $7,232,881 | 0% | 9,202 | 9,202 | 0% |
| Voluntary medical male circumcision (VMMC) | $6,374,449 | $5,766,208 | -10% | 17,884 | 43,942 | 145% |
| Condom programs | $2,893,393 | $3,914,852 | 35% | 427,981 | 487,500 | 14% |
| HIV prevention and testing programs targeting female sex workers (FSW) | $573,413 | $719,794 | 26% | 3,199 | 3,399 | 6% |
| Antiretroviral-based prophylaxis  (PEP, PrEP) | $78,840 | $533,571 | 577% | 396 | 22,880 | 5,675% |
| Text messaging adherence support | $0 | $140,154 | Increased | 0 | 107,392 | Increased |
| Efforts to keep girls in school | $61,083 | $118,275 | 94% | 2,002 | 3,873 | 93% |
| Text messaging appointment reminder | $6,160 | $92,525 | 1,402% | 19,450 | 146,790 | 655% |
| Enhanced adherence counselling | $2,247 | $62,590 | 2,685% | 1,170 | 31,976 | 2,632% |
| Linkage to care - appointment support (escorted/met for appointment/transport) | $2,090 | $53,628 | 2,466% | 529 | 12,825 | 2,324% |
| Social and behavior change communication (SBCC) | $4,226,272 | $0 | -100% | 285,280 | 0 | -100% |
| HIV prevention and testing programs targeting men who have sex with men (MSM) | $155,140 | $0 | -100% | 821 | 0 | -100% |
| Linkage to care - telephone follow-up | $1,140 | $0 | -100% | 545 | 0 | -100% |
| Pre-ART tracing missed appointments | $132,170 | $0 | -100% | 43,453 | 0 | -100% |

Source: Optima HIV model, 2018
