# Supplementary material for: Opportunities for improved HIV prevention and treatment through budget optimization in Eswatini
Source: PLoS One. 2020 Jul 23;15(7):e0235664. doi: 10.1371/journal.pone.0235664 (PMC7377429; doi:10.1371/journal.pone.0235664)
Supplement: S6 Table — (DOCX) [file pone.0235664.s009.docx]

Table S6. Optimised allocation of varying budget levels

| **HIV programs** | **Optimised 50% budget** | **Optimised 90% budget** | **Baseline budget** | **Optimised 100% budget** | **Optimised 110% budget** | **Optimised 150% budget** | **Optimised 200% budget** |
| --- | --- | --- | --- | --- | --- | --- | --- |
| Antiretroviral therapy (ART) | $29,109,140 | $39,340,595 | $39,521,381 | $39,521,381 | $39,521,381 | $39,948,937 | $40,030,868 |
| HIV testing services (HTS) | $0 | $3,982,413 | $4,190,502 | $7,057,973 | $8,000,447 | $12,008,469 | $12,356,833 |
| Prevention of mother-to-child transmission (PMTCT) | $3,616,441 | $6,592,591 | $7,232,881 | $7,232,881 | $7,956,169 | $10,849,322 | $14,465,762 |
| Voluntary medical male circumcision (VMMC) | $0 | $4,598,450 | $6,374,449 | $6,193,112 | $6,991,167 | $8,775,873 | $26,590,938 |
| Condom programs | $0 | $3,521,325 | $2,893,393 | $4,322,701 | $5,452,349 | $5,457,824 | $6,418,960 |
| HIV prevention and testing programs targeting female sex workers (FSW) | $0 | $689,874 | $573,413 | $629,469 | $658,512 | $1,063,875 | $596,319 |
| Antiretroviral-based prophylaxis (PEP, PrEP) | $0 | $0 | $78,840 | $3,797 | $3,197 | $4,131,407 | $4,437,281 |
| Text messaging adherence | $0 | $73,932 | $0 | $154,099 | $136,326 | $292,834 | $335,529 |
| Efforts to keep girls in school | $0 | $0 | $61,083 | $70,861 | $553,846 | $2,174,816 | $2,714,005 |
| Text messaging appointment reminder | $0 | $105,863 | $6,160 | $106,745 | $110,596 | $152,985 | $166,233 |
| Enhanced adherence counselling | $0 | $0 | $2,247 | $582 | $173,956 | $454,676 | $496,172 |
| Linkage to care - appointment support (escorted/met for appointment/transport) | $0 | $0 | $2,090 | $51,721 | $1,817 | $177,573 | $198,422 |
| Social and behaviour change communication (SBCC) | $0 | $0 | $4,226,272 | $105,152 | $2,434,936 | $11,916,440 | $21,131,209 |
| HIV prevention and testing programs targeting men who have sex with men (MSM) | $0 | $0 | $155,140 | $0 | $0 | $0 | $65,916 |
| Linkage to care - telephone follow-up | $0 | $1,003 | $1,140 | $687 | $1,578 | $112,723 | $129,286 |
| Tracing missed appointments | $0 | $0 | $132,170 | $0 | $0 | $658,986 | $768,588 |

Source: Optima HIV model, 2018

**References**

1. Forsythe, S. Evaluating the cost-effectiveness and impact of oral pre-exposure prophylaxis for HIV prevention in Eswatini. AIDS conference 2018: Amsterdam, Netherlands.

2. National Emergency Response Council on HIV and AIDS. The extended National Multisectoral HIV and AIDS Framework. 2014: Manzini, Swaziland.

3. Kingdom of Eswatini Ministry of Health. Swaziland male circumcision strategic and operational plan for HIV prevention, Swaziland National AIDS Program. 2014: Mbabane, Swaziland.

4. Kingdom of Eswatini Ministry of Health. MaxART early access to ART for all implementation study (2014-2018) Final Report. 2018: Mbabane, Ewatini.

5. MEASURE Evaluation. The costs of HIV treatment, care, and support services in Uganda, PEPFAR. 2013: Washington DC, USA.
