# Supplementary material for: Opportunities for improved HIV prevention and treatment through budget optimization in Eswatini
Source: PLoS One. 2020 Jul 23;15(7):e0235664. doi: 10.1371/journal.pone.0235664 (PMC7377429; doi:10.1371/journal.pone.0235664)
Supplement: Attachment — Submitted filename: Eswatini_review.docx [file pone.0235664.s010.docx]

Review of the manuscript “Opportunities for improved HIV prevention through budget optimisation in Eswatini”.

General comments:

The writing of the manuscript could be improved. There are sentences that are not very well structured throughout the manuscript.

I think the manuscript would be strengthened if it does not focus on opportunities alone but also includes the challenges. The title could be “Opportunities and challenges for improved HIV prevention and treatment through budget optimization in Eswatini”.

The title does not mention “treatment”, but the manuscript does include optimization of different ART care modalities. I would therefore suggest to add “treatment” to the title. See suggested title above.

In order to evaluate the validity of the study, I would like a bit more information about the Optima model and optimization functions used in the manuscript.

I am confused about the Table 1 and 2 strategies. Would it not make more sense to find the most optimal strategy among the HIV testing modalities and ART care modalities first in separate (competing choice) analyses and put the most cost-effective option in your optimization list of strategies instead of finding/optimizing the budget for e.g., treatment and then reallocating funds to the more cost-effective strategies within HIV testing modalities and ART care modalities? Currently I am not sure how you have calculated costs and effect for e.g. treatment if you do not know which ART care modality has been used (because you find the most cost-effective options after treatment budget determination).

More specific comments:

- 1^st^ sentence Introductions: please indicate the number of new infections per year. For example: “Since 2014, substantial scale-up of HIV testing and treatment in Eswatini has led to a 44% decrease in new infections from xxxx/1000 adults to xxxx/1000 adults annually.
- The last sentence of the 1^st^ paragraph of the Introduction section should be rephrased, because the sentence is not correct as it is.
- 2^nd^ sentence 2^nd^ paragraph of the Introduction: I would mention that it concerns the HIV budget by rephrasing the sentence to “In line with ….. 25% of the total HIV budget on prevention, the Eswatini government …….”.
- I would clarify that the NSF period is 5 years in the last sentence of the 2^nd^ paragraph of the Introduction: “The proposed target was a 85% reduction in new infections by the end of the 5-year NSF period (2018-2023).”
- Can the authors add to the manuscript how the current NSF is aiming/ proposes to accomplish a 85% reduction in new HIV infections.
- I would be interested in what the current HIV budget is in Eswatini. Can the authors mention that in the Introduction?
- The authors mention that allocative efficiency modeling has taken place already, but it was unclear if this type of analysis was integrated in the 2018-2023 NSF. Can the authors make this more explicit in the text?
- It was unclear whether the analysis of the current study serves to inform a strategic document. The NSF has already been published and only ends in 2023. Can the authors clarify this? It seems like a redundant analysis if the NSF 2018-2023 has already been informed by an allocative efficiency analysis. In other words, please make clear how this analysis fits in/ add to the analysis already conducted and for which strategic document (if any) this analysis has been done.
- The authors state “Recommendations from these studies have been adopted by the country Government, and the current response may already be highly allocatively efficient.” This statement is rather vague. Do we not know? I would be interested in the degree to which Eswatini has implemented the results of this allocative efficiency analysis that has been undertaken. Can the authors mention anything about that in the text?

Methods section

- What is the budget that the authors are working with in their optimization analysis?
- Can the authors elaborate a bit more (in a couple of sentences) on the Optima HIV model for the readers who do not know the model. The information currently provided is very scarce. I am not sure what the authors mean with a compartmental model. Also, it would be good to mention that the Optima model is a commonly-used model in costing and optimization exercises for strategic documents with some references of analyses in which the Optima model has been used.
- Can the authors be more elaborate in the data sources section. The paragraph before that states that a prior model analysis has been updated. In the data sources section, I would be interested in what data has been updated and how for the new model.
- In the model calibration section, it was stated that estimates were calibrated to estimates from other models, but no references were provided here. To what other models were the estimates calibrated to?
- In the analysis approach section: how were cost functions updated? And from what? From the optimization algorithm described elsewhere? Can you add a couple of sentences describing the methodology behind the algorithm? Its hard to evaluate the validity of a study when the most crucial elements such as the optimization algorithm is not described in the current manuscript.
- In the “impact of optimized allocative efficiency” section, please mention what the currently available budget is.
- Is is unclear whether implementation costs are also considered in the program costs.
- The two strategies mentioned just before the Results section (a regimen of DTG for non-pregnant adults and VMMCs) came as a surprise. If these are considered strategies in the optimization exercise these should be mentioned in the table of available treatment and prevention strategies. Now it comes across as just trying out some things.

Results section

- It is unclear whether the strategies listed at the end of the 1^st^ paragraph of the results section are just included in the optimization exercise or actually the chosen programs to fund based on the budget. A clarification would help. As well as a table with all strategies/programs considered for funding.
- 2^nd^ paragraph of the results section: please specify “a large increase in infections and deaths” by mentioning the actual number of infections and number of deaths for the budget cut of 50% or the % of increase for these 2 outcomes.
- The results section mentions: “At higher optimised budget levels of 150% and 200%, after the maximum number of people are tested in a given year, further infections may be averted by prioritizing primary prevention programs that target the general population such as VMMC, prevention of mother-to-child transmission (PMTCT), programs to keep girls in school, ARV-based prophylaxis, social and behaviour change communication (SBCC), and to a lesser extent condom programs.”

What do you mean with “may”? Are you guessing or are these the programs that should be funded based on the optimization exercise?

- Page 10, last paragraph: do you mean 2023 instead of 2022?
- The Results section states: “Due to a lower unit cost, public fixed or integrated VMMC sites (over mobile VMMC sites) are likely more efficient.” If this is not a result of the analysis, mention it in the Discussion section instead.
- The 2 unit costs mentioned in the last paragraph either need to be accompanied by a source reference or an explanation of how the unit cost was estimated and what exact components it consists of.

Discussion section

- 1^st^ paragraph: “HIV testing amongst the general population should be scaled up to diagnose more people ….”. How much more? How much scale up? Be more precise here. What type of testing you are talking about (from Table 1)?
- Have you taken into account that Eswatini has started implementing prior optimization results already? If so, how?
- I am confused (2^nd^ paragraph): are we not talking about the 2018-2023 NSF timeframe mentioned earlier?
- They won’t make the projected 85% reduction. Mention what they make instead in the 2^nd^ paragraph.
- “Exploring the possibility for switching non-pregnant adults on stable treatment to lower cost ARV drugs, such as Dolutegravir-based regimens, may result in cost savings”. What do you mean with “may”? You have investigated that in this study and should now know the answer.
- “Although the model optimisation algorithm accounts for inherent uncertainty, it might not be possible to account for all aspects of uncertainty because of poor quality or insufficient data, particularly for important cost values.” What do the authors mean with inherent uncertainty? I am missing sensitivity analyses for the most important cost values. This seems important as the quality of the cost data used is difficult to evaluate.
- In the conclusion section I would not mention the “If additional funding can be secured …” as that is an unrealistic scenario in the light of budget cuts. I would focus on the scenario of budget reduction and the optimal spending of the current budget.
