# Supplementary material for: Opportunities for improved HIV prevention and treatment through budget optimization in Eswatini
Source: PLoS One. 2020 Jul 23;15(7):e0235664. doi: 10.1371/journal.pone.0235664 (PMC7377429; doi:10.1371/journal.pone.0235664)
Supplement: Attachment — Submitted filename: Eswatini_reviewer response.docx [file pone.0235664.s011.docx]

| **Editor comments** | **Author response** | **Changes to manuscript where relevant** |
| --- | --- | --- |
| 1. Please ensure that you refer to Figure 2 in your text as, if accepted, production will need this reference to link the reader to the figure. | Figure 2 has now been referenced in the second paragraph of the Results section. |  |
| 2. Please include captions for your Supporting Information files at the end of your manuscript, and update any in the text citations to match accordingly. | Descriptive captions have been added in the Supplementary information. |  |

Dear PLOS One Editor,

## The authors of the manuscript ‘Opportunities for improved HIV prevention and treatment through budget optimisation in Eswatini’ [PONE-D-19-25759] [MID:ba465f85d9ae709a] would like to thank you and the reviewers for effort spent reviewing our manuscript. The comments are very useful and have helped us strengthen the manuscript. We have addressed all comments, as shown in the table below, as well as in the revised manuscript

Please feel free to contact use should you need any further clarification or revisions.

Kind regards,

Sherrie

Sherrie Kelly, corresponding author on behalf of all authors

| **Reviewer #1** | **Author response** | **Changes to manuscript where relevant** |
| --- | --- | --- |
| **General comments** |  |  |
| The writing of the manuscript could be improved. There are sentences that are not very well structured throughout the manuscript. | We thank the reviewer for their comments, the writing has been thoroughly reviewed and tightened throughout. |  |
| I think the manuscript would be strengthened if it does not focus on opportunities alone but also includes the challenges. The title could be “Opportunities and challenges for improved HIV prevention and treatment through budget optimization in Eswatini”. | We thank the reviewer for the interesting and relevant suggestion. We did not explore the specific challenges. Therefore, it would be difficult to include them as it would involve further data collation and expansion of scope. As such, we have not included this in the title, but will certainly keep in mind for future work. |  |
| The title does not mention “treatment”, but the manuscript does include optimization of different ART care modalities. I would therefore suggest to add “treatment” to the title. See suggested title above. | We thank the reviewer for this suggestion. ‘Treatment’ has been added to the title. | Title now reads: Opportunities for improved HIV prevention and treatment through budget optimisation in Eswatini |
| In order to evaluate the validity of the study, I would like a bit more information about the Optima model and optimization functions used in the manuscript. | More information about the Optima model has been added to the Methods in the manuscript, as well as described in the methods section below. | The following has been added to paragraph one of the Methods, “The optimisation algorithm applies adaptive stochastic descent to calculate the optimal allocation of a finite set of resources against defined constrained objective functions. The algorithm forms probabilistic assumptions about which parameters, such as changes in spending on programmes which influence prevention, treatment, and other outcomes, have the greatest effect on minimising new infections and uses optimal step sizes for each parameter (12).” |
| I am confused about the Table 1 and 2 strategies. Would it not make more sense to find the most optimal strategy among the HIV testing modalities and ART care modalities first in separate (competing choice) analyses and put the most cost-effective option in your optimization list of strategies instead of finding/optimizing the budget for e.g., treatment and then reallocating funds to the more cost-effective strategies within HIV testing modalities and ART care modalities? Currently I am not sure how you have calculated costs and effect for e.g. treatment if you do not know which ART care modality has been used (because you find the most cost-effective options after treatment budget determination). | The reviewer has suggested an interesting approach; however, since the testing and ART modalities are targeted at different populations, it would therefore be sub-optimal to not include certain modalities in the final optimization analysis. Instead the total optimized budget for core programs has been derived. This budget has then been applied across the different modalities (individually for treatment and testing). The modalities which either minimize HIV related deaths and new infections under the same budget have been described, or which minimize budget with the same number of infections and deaths averted have been described. In the case of the latter, this represents further budget to be re-optimised in the general analysis. This has been clarified in the text. | The following has been added to the *analysis approach* section: The analysis approach was to first optimise the current budget for big bucket items such as standard ART. Following this, sub-analysis of implementation modalities for treatment, and testing were performed to either minimise cost, or minimise new HIV infections and deaths. Modalities differed in their target populations and cost-functions. |
| **More specific comments** |  |  |
| 1^st^ sentence Introductions: please indicate the number of new infections per year. For example: “Since 2014, substantial scale-up of HIV testing and treatment in Eswatini has led to a 44% decrease in new infections from xxxx/1000 adults to xxxx/1000 adults annually. | Thank you for the addition this has been amended in text |  |
| The last sentence of the 1^st^ paragraph of the Introduction section should be rephrased, because the sentence is not correct as it is. | Thank you, this sentence has been revised. | This sentence now reads: Despite encouraging progress, HIV remains Eswatini’s leading cause of disability and death (4), with one the highest incidence of HIV in the world (1). |
| 2^nd^ sentence 2^nd^ paragraph of the Introduction: I would mention that it concerns the HIV budget by rephrasing the sentence to “In line with ….. 25% of the total HIV budget on prevention, the Eswatini government …….”. | This sentence already describes the modelling exercise as incorporating the HIV budget. More clarity is needed to address this comment. |  |
| I would clarify that the NSF period is 5 years in the last sentence of the 2^nd^ paragraph of the Introduction: “The proposed target was a 85% reduction in new infections by the end of the 5-year NSF period (2018-2023).” | Thank you, this has been altered in the text. |  |
| Can the authors add to the manuscript how the current NSF is aiming/ proposes to accomplish a 85% reduction in new HIV infections. | It is beyond the scope of this manuscript to provide an in-depth analysis of the NSF, however, an overview of the strategies stated in the NSF have been added to the manuscript. | The following has been added to the second paragraph (newly added section in italics): The proposed target was an 85% reduction in new infections by the end of the 5-year NSF period (2018-2023). *This is to be achieved by implementing selected core interventions including combination HIV prevention strategies, scaling up treatment and care services, and strengthen cross-cutting areas to create an enabling environment for improving gender equity and women’s empowerment.* (7). |
| I would be interested in what the current HIV budget is in Eswatini. Can the authors mention that in the Introduction? | Thank you for this addition, the total budget, along with the budget available for optimization has been added to the last paragraph of the introduction. | The following has been added to the last paragraph of the introduction: This was achieved by modelling optimised allocation of ESwatini’s most recent available national annual HIV budget of $123 million USD (2018), of which $63 million was devoted to targeted programs whose direct impact could be modelled. |
| The authors mention that allocative efficiency modeling has taken place already, but it was unclear if this type of analysis was integrated in the 2018-2023 NSF. Can the authors make this more explicit in the text? | The current study is part of a study designed to support the development of the NSF, the process of which has been ongoing during the finalization of the NSF. This article is also designed to help inform cost-effective approaches to reaching the NSF strategic goals. It is important however, to understand that this project provides only recommendations which may not necessarily be incorporated into strategic planning. This has been clarified in text. | The final paragraph of the introduction now reads: In the face of potential funding cuts, we explored approaches to improve the cost-effectiveness of HIV prevention and treatment in Eswatini through a modelling analysis in line with NSF objectives |
| It was unclear whether the analysis of the current study serves to inform a strategic document. The NSF has already been published and only ends in 2023. Can the authors clarify this? It seems like a redundant analysis if the NSF 2018-2023 has already been informed by an allocative efficiency analysis. In other words, please make clear how this analysis fits in/ add to the analysis already conducted and for which strategic document (if any) this analysis has been done. | Please see previous comment which also pertains to this question. |  |
| The authors state “Recommendations from these studies have been adopted by the country Government, and the current response may already be highly allocatively efficient.” This statement is rather vague. Do we not know? I would be interested in the degree to which Eswatini has implemented the results of this allocative efficiency analysis that has been undertaken. Can the authors mention anything about that in the text? | The previous iteration of the modelling exercise was conducted prior to the NSF, with the intention to provide analytical support to an HIV investment case in Eswatini. This has been clarified in the text. The current model was constructed during the NSF construction process and as such has been used to provide recommendations during the NSF process. As per the previous comment however, these are recommendations and are not necessarily incorporated into strategic planning. | The relevant section now reads: Allocative efficiency modelling analysis, conducted previously in Eswatini have demonstrated that substantial gains may be possible by better allocating financial resources across the current mix of broad HIV program categories, such as HIV testing or treatment (9). Recommendations from these studies were taken into account by the country Government and Ministry of Health as part of the HIV strategic planning process. |
| **Methods section** |  |  |
| What is the budget that the authors are working with in their optimization analysis? | The annual national budget is $127 million total, of which $63 million can be optimized. This has been added to the first sentence of the introduction as per the reviewer comment above. |  |
| Can the authors elaborate a bit more (in a couple of sentences) on the Optima HIV model for the readers who do not know the model. The information currently provided is very scarce. I am not sure what the authors mean with a compartmental model. Also, it would be good to mention that the Optima model is a commonly-used model in costing and optimization exercises for strategic documents with some references of analyses in which the Optima model has been used. | The first paragraph of the methods section now describes the Optima HIV model in more detail. The reader has also been pointed to the Optima HIV website which lists several examples of where Optima HIV has been implemented, as well as associated application reports and study publications. | First paragraph now reads: Optima HIV (hiv.optimamodel.com, version 2.6.11), a dynamic population-based compartmental model, was applied (12). This model tracks HIV transmission and is overlaid with a programmatic costing component and optimisation algorithm. The compartmental module tracks HIV dynamics between population groups, such as females 15-24, or female sex workers (FSW), over time incorporating assumptions around interactions between populations, transmissibility of HIV, and behavioural parameters. This is overlaid with a module incorporating the effect of programs targeted at reducing new HIV infections, HIV related morbidity, and HIV related death such as antiretroviral treatment (ART) or condom programs. An individual programs coverage can be altered through its unit costs and total budget. The optimisation algorithm applies adaptive stochastic descent to calculate the optimal allocation of a finite set of resources devoted to each program against defined constrained objective functions, such as the relative weighting of reducing new HIV infections or preventing HIV-related deaths. The algorithm forms probabilistic assumptions about which parameters, such as changes in spending on programmes which influence prevention, treatment, and other outcomes, have the greatest effect on minimising the objective function and uses optimal step sizes for each parameter (12).  Optima HIV is a commonly used model in costing and optimisation exercises for national and sub-national HIV strategy development. Various examples of Optima HIV being used to help inform evidence-based HIV strategy can be found on the Optima website (hiv.optimamodel.com). |
| Can the authors be more elaborate in the data sources section. The paragraph before that states that a prior model analysis has been updated. In the data sources section, I would be interested in what data has been updated and how for the new model. | The data sources used to inform the Optima HIV model are extensive, and as such have been provided in detail in the supplementary materials section. The use of data drawn from the previous modelling exercise has been clarified in the *Data sources* section of the methods. | The following has been added to the data sources section of the methods: The model used in this analysis builds upon a previous modelling exercise for Eswatini completed in 2014. |
| In the analysis approach section: how were cost functions updated? And from what? From the optimization algorithm described elsewhere? Can you add a couple of sentences describing the methodology behind the algorithm? Its hard to evaluate the validity of a study when the most crucial elements such as the optimization algorithm is not described in the current manuscript. | Cost functions have been updated from the previous modeling exercise conducted for Eswatini to reflect more recent cost and coverage data, with ad. | Additional text has been added to further describe the optimization algorithm in the first paragraph of the Methods section. |
| In the “impact of optimized allocative efficiency” section, please mention what the currently available budget is. | This has been added to the relevant section. | The following has been added to the *impact of optimized allocative efficiency section:* This was designed to estimate an optimised mix of programs under the constraint of the most recent available budget of $123 million, of which $63 million was able to be optimised. |
| It is unclear whether implementation costs are also considered in the program costs. | This depends on which implementation cost is being considered. The building of infrastructure, such as hospitals, is not included in the analysis, management costs etc. where relevant are included in the non-targeted program costs section. This is true except for programs which are explicitly measuring the reduction in cost associated with activities such as integration – as is the case for the VMMC sub-analysis. In this case, the cost difference is discussed in the relevant section. This has been clarified in the text. | The following has been added to the *analysis approach* section of the methods: Implementation costs, such as building of infrastructure, are either not included in the analysis or incorporated into non-targeted program costs unless specified. |
| The two strategies mentioned just before the Results section (a regimen of DTG for non-pregnant adults and VMMCs) came as a surprise. If these are considered strategies in the optimization exercise these should be mentioned in the table of available treatment and prevention strategies. Now it comes across as just trying out some things. | These two strategies differ from those mentioned in the tables as they have the same effectiveness and the same populations were targeted as for the mainstream ART refill option, similarly for the VMMC modality analysis, but differ in unit cost. Therefore, it is not possible to compare these to other treatment modalities. These analyses are only to estimate their impact. As for two programs with the same effectiveness, but different unit cost, the program with the lower cost will be prioritized. The purpose of these analyses has been clarified in the text. | Added to second last paragraph of methods: This was performed as an additional analysis as the effectiveness of DTG was assumed to be the same as the standard current ART regimen.  Added to last paragraph of methods: This was also performed as an additional analysis as the effectiveness of VMMC under this scenario did not differ from standard VMMC. |
| **Results section** |  |  |
| It is unclear whether the strategies listed at the end of the 1^st^ paragraph of the results section are just included in the optimization exercise or actually the chosen programs to fund based on the budget. A clarification would help. As well as a table with all strategies/programs considered for funding. | These are included in the list of recommended programs based on the optimization. They are already described in figure 1. This has been clarified in the text. | Final sentence of paragraph 1 of results now reads: These include efforts to keep girls in school, condom distribution, VMMC, FSW non-ART prevention programs, and ARV-based prophylaxis (namely pre- and post-exposure prophylaxis respectively) as recommended based on the optimisation analysis seen in figure 1. |
| 2^nd^ paragraph of the results section: please specify “a large increase in infections and deaths” by mentioning the actual number of infections and number of deaths for the budget cut of 50% or the % of increase for these 2 outcomes. | The total number has been added in text. | The sentence now reads: If the HIV budget were to be reduced by 50%, even under optimised allocation, not unexpectedly a large increase in infections and deaths was projected (2019-2023 cumulatively 170% more new infections and 40% more HIV-related deaths than baseline) (figure 2), deprioritising all programs other than treatment (ART and PMTCT). |
| The results section mentions: “At higher optimised budget levels of 150% and 200%, after the maximum number of people are tested in a given year, further infections may be averted by prioritizing primary prevention programs that target the general population such as VMMC, prevention of mother-to-child transmission (PMTCT), programs to keep girls in school, ARV-based prophylaxis, social and behaviour change communication (SBCC), and to a lesser extent condom programs.”  What do you mean with “may”? Are you guessing or are these the programs that should be funded based on the optimization exercise? | The use of ‘may’ is to represent that the analysis is a modelling exercise and may not necessarily reflect the reality on the ground if these initiatives were implemented. This has been altered to ‘can’. |  |
| Page 10, last paragraph: do you mean 2023 instead of 2022? | Yes, thank you for this clarification. This has been altered. |  |
| The Results section states: “Due to a lower unit cost, public fixed or integrated VMMC sites (over mobile VMMC sites) are likely more efficient.” If this is not a result of the analysis, mention it in the Discussion section instead. | This has been added to the discussion section. | Paragraph 5 of the discussion section now reads: Finally, savings may be found through integration of service delivery and other approaches for health system strengthening that can leverage pre-existing infrastructure such as VMMC delivered at fixed sites (19), as lower unit cost, public fixed or integrated VMMC sites (over mobile VMMC sites) are likely more efficient. |
| The 2 unit costs mentioned in the last paragraph either need to be accompanied by a source reference or an explanation of how the unit cost was estimated and what exact components it consists of. | Both unit costs have a description in the text, both are drawn from country partners, and therefore do not have a specific reference. These unit costs are used for demonstrative purposes only in the sub-analyses. |  |
| **Discussion section** |  |  |
| 1^st^ paragraph: “HIV testing amongst the general population should be scaled up to diagnose more people ….”. How much more? How much scale up? Be more precise here. What type of testing you are talking about (from Table 1)? | The information on the magnitude of scale-up is available in figure 1. We generally avoid explicitly stating exact dollar amounts or % scale up in the main text as it conveys an over-confidence in the modelled results. |  |
| Have you taken into account that Eswatini has started implementing prior optimization results already? If so, how? | We have taken into account how Eswatini has likely implemented prior optimization results in the 4^th^ paragraph of the introduction. We avoid explicitly stating that they are implementing optimization analysis as our support are provided only as recommendations. |  |
| I am confused (2^nd^ paragraph): are we not talking about the 2018-2023 NSF timeframe mentioned earlier? | This paragraph mentions the 2023 timeline. More details are needed to address this comment. |  |
| They won’t make the projected 85% reduction. Mention what they make instead in the 2^nd^ paragraph. | We avoid explicitly stating in the main text the exact results of the different modelling scenarios as it can display an overconfidence in the modelled results. |  |
| “Exploring the possibility for switching non-pregnant adults on stable treatment to lower cost ARV drugs, such as Dolutegravir-based regimens, may result in cost savings”. What do you mean with “may”? You have investigated that in this study and should now know the answer. | Following the comment above the word ‘may’ has been used to indicate that this analysis is a modelling study and may not necessarily reflect the real-world situation. This work could be altered to ‘can’, but the authors feel we do not have sufficient certainty to do so. |  |
| “Although the model optimisation algorithm accounts for inherent uncertainty, it might not be possible to account for all aspects of uncertainty because of poor quality or insufficient data, particularly for important cost values.” What do the authors mean with inherent uncertainty? I am missing sensitivity analyses for the most important cost values. This seems important as the quality of the cost data used is difficult to evaluate. | The Optima HIV model is set-up to run sensitivity analysis for cost by permutating through a range of unit costs for each HIV program (along with other factors used in sensitivity analyses). |  |
| In the conclusion section I would not mention the “If additional funding can be secured …” as that is an unrealistic scenario in the light of budget cuts. I would focus on the scenario of budget reduction and the optimal spending of the current budget. | In the Conclusion it is noted that additional funding may be found through expansion of the existing budget or through increased efficiency with the existing budget. The latter is demonstrated in our analysis. We have clarified this in text. | Conclusions section now reads: If additional funding can be secured, either through *the unlikely scenario of* expansion of the existing budget or *more likely through* finding efficiency in the implementation of existing programs, optimally investing in established cost effective primary prevention programs will help to further reduce new infections in the country. |
|  |  |  |

| **Reviewer #2** | **Author response** | **Changes to manuscript where relevant** |
| --- | --- | --- |
| **General comment** |  |  |
| Overall, I think this is an interesting and important topic. It addressed an urgent issue in the HIV prevention in Eswatini. The findings could be used to inform and improve the current HIV practice. I have some specific suggestions below. Some expert opinions are needed for the modeling. I assumed that the modeling part was conducted properly for analyzing the questions in this paper. | We thank the reviewer for their time in reviewing the manuscript and interest in our study. The reviewer comments have helped strengthen the manuscript and provide a more cohesive argument for the use of modelling in HIV strategy. |  |
| **Major comments** |  |  |
| 1. For Table 1, please provide what was the targeted population in each priority implementation site. For Table 1 and Table 2, please provide what the current percent coverage was for each HIV testing modality and each ART modality in the overall population. | We thank the reviewer for these comments; however, modalities are more targeted towards implementation sites, rather than specific populations. It will not be of added benefit to the reader to see for example that in rural sites, interventions are targeted at rural PLHIV.  Regarding coverage, this information is already provided in figure 3 (the right most bar of the bar graph). |  |
| 2. On page 8, “measured the effect that switching eligible people to those modalities had on cost, coverage, retention, and viral suppression”. Were the implementation/switching costs considered or included in the analyses besides the program costs? Please explain this point in the paper. | Implementation costs for the various modalities were considered where relevant, such as the cost of transport for outreach ART. General implementation costs however, were not included in the analysis (for example the cost of building hospitals), or included in the cost of non-targeted programs (such as general management or HR). This has been clarified in the text. | The *analysis approach* section of the methods now reads: Implementation costs, such as building of infrastructure, are either not included in the analysis or incorporated into non-targeted program costs unless specified. |
| 3. On page 10, “A budget incorporating VMMC with reduced start-up costs and other implementation efficiencies results in an average unit cost of $140 (estimated unit cost cited by the country for planning purposes and other bottom-up costing analyses) compared to $364 at baseline (estimated using a top-down costing approach using data for baseline coverage and expenditure including start-up costs obtained from implementing partners).” Are the unit costs of two scenarios comparable by using two different methods? Please justify this. | Thank you for this comment. This analysis represents the potential difference which removing start up-costs from VMMC could produce. The second unit cost can be thought of as the current or actual unit cost. The first unit cost is devised by country experts to demonstrate the potential impact of removing start-up costs. As stated in the text the first unit cost is devised based on country expert opinion. This has been clarified in text. |  |
| 4. In the discussion session (page 12), “Our findings show that implementation efficiencies can be actualized by optimising across cost-effective modalities.” What is the feasibility consideration regarding increasing/decreasing funding for different testing and ART modalities? How will the changes impact different groups of population, such as rural population vs. urban population, etc.? Please provider some discussion on this. | Our model does not readily incorporate differing access or costs associated with urban and rural populations. Primarily because these differences would be difficult to accurately parameterize for the different interventions/programs. This point has been added with further clarification in the text. | The following has been added to paragraph 4 of the discussion: These recommendations, however, should be balanced with context specific considerations not incorporated into the modelling approach such as access and cost differences associated with delivering ART in urban and rural locations. |
| 5. In the discussion session (page 12), “Community-based ART groups reduce stress on both the healthcare system and on people needing treatment refills by allowing multiple patients to share the logistic burden of collecting their drug refills.” How would this change impact adherence? Are there studies providing evidence on this? | The authors are not claiming a link between community-based ART groups and adherence necessarily, simply stating that the community-based model allows for the possibility of reduced financial burden on the system and patients. This has been clarified in text. | Now reads: Community-based ART groups reduce stress on the healthcare system by allowing multiple patients to share the logistic burden of collecting their drug refills. |
| 6. Authors mentioned several limitations in the discussion session. One of them is that “Although the model optimisation algorithm accounts for inherent uncertainty, it might not be possible to account for all aspects of uncertainty because of poor quality or insufficient data, particularly for important cost values.” To what extent, the findings would still hold under this limitation. Have you done sensitivity analysis to explore some of those data uncertainties? | The level to which results findings still hold under this limitation are inherently subjective. Optima provides a likely direction of results if certain conditions (or assumptions) are met, as with any modelling exercise. The authors have stressed throughout the manuscript that results are estimates and to be treated with caution.  In relation to sensitivity analyses, Optima inherently incorporates sensitivity of cost by utilizing a range of unit costs for each program. A full sensitivity analysis is not pursued as the number of parameters used in Optima make providing meaningful sensitivity bounds impossible. |  |
| 7. In the discussion, authors mentioned the study was from a provider perspective. Please mention this in the method section and justify or explain the reasons for pick this perspective. | This has been added to the methods section. | The following has been added to the *analysis approach* section of the methods: Costs incorporated into the model are included from a provider perspective, as the analysis is conducted primarily to inform national strategic decision making. While highly relevant, out of pocket costs incurred by people accessing the HIV program are not included. |
| 8. For figure 2, except scenario “Optimized 50%”, other scenarios had similar trends and results regarding the outcome, new HIV infections. It would be helpful to inform how new HIV infections change in scenarios when Optimized between 50% and 90%. This would help to show what the critical points/cutoffs are for the trends and results to change. | To clarify the number differences between the different optimisation scenarios, the differences between 50% and 90% optimized budgets have been added. | An optimised 50% budget is also estimated to cumulatively result in 160% more new infections and 40% more HIV-related deaths than a 90% optimised budget. |
| **Minor points** |  |  |
| 9. On page 9, “If the budget were cut by 10% (to 90% of the latest reported amount), but with optimised allocation, an estimated 300 more new HIV infections (1% more) and 100 more HIV-related deaths (less than 1% more) could be averted by 2023. Optimised allocation of budgets increased to 110%, 150% and 200% are estimated to result in 7%, 15% and 18% fewer total new infections by 2023, respectively, showing that return on investment marginally decreases with increasing budget at these levels. With an increase to 110% optimised budget, HIV testing should be prioritized.”  In the first sentence, both the number of cases and the percent increase were mentioned for both HIV-related deaths and new HIV infections. In the second sentence, only the percent decrease in new infections were mentioned. Please be consistent and add in number of cases increased/decreased for both outcomes in the second sentence. | To avoid overburdening this sentence, the total number of infections and deaths have been removed. There is now consistency in how the data is presented. |  |
| 10. For figure 3, besides the number of people in the y-axis, please also provide the coverage in percent of total population. | Presenting the coverage in this way may be misleading to the reader as different testing modalities are targeted at different populations, thus denominators for each testing modality will differ. |  |
| 11. Please be clear about what saturation means in the supporting information. It is not completely clear to the general audience for this journal | This point is highly relevant to the general manuscript and has been clarified in the main text. | Cost functions also incorporate saturation, which is the increasing marginal cost associated with covering higher proportions of a targeted population. A cost function is considered saturated at the point where further increases to the budget will no longer result in increased coverage. This is analogous to the increasing difficulty in reaching the last hardest to reach individuals in a population. |
