# Supplementary material for: Opportunities for improved HIV prevention and treatment through budget optimization in Eswatini
Source: PLoS One. 2020 Jul 23;15(7):e0235664. doi: 10.1371/journal.pone.0235664 (PMC7377429; doi:10.1371/journal.pone.0235664)
Supplement: Attachment — Submitted filename: Eswatini HIV second response to reviewers.docx [file pone.0235664.s012.docx]

Dear PLOS One Editor,

## The authors of the manuscript ‘Opportunities for improved HIV prevention and treatment through budget optimisation in Eswatini’ [PONE-D-19-25759] [MID:ba465f85d9ae709a] would like to thank you and the reviewer for effort spent reviewing our manuscript for a second time. The comments are very useful and have helped us strengthen the manuscript. We have addressed all comments, as shown in the table below, as well as in the revised manuscript

Please feel free to contact use should you need any further clarification or revisions.

Kind regards,

Sherrie

Sherrie Kelly, corresponding author on behalf of all authors

| **Reviewer #1** | **Author response** | **Changes to manuscript where relevant** |
| --- | --- | --- |
| **Major comments** |  |  |
| Major comment: Were there any challenges in obtaining the data needed to populate the model? The major problem with this type of analyses is that high-quality unit cost data is often not available. Because of this challenge, comparison of actual trends in HIV/AIDS spending and program output against resource needs estimates that had been calculated for strategic planning reveal large variances. That variation is problematic in planning. The authors mention the quality of the cost data in the limitations, but as a reader (who appraises the validity of the study findings while reading) I would like to see in the Methods section what the sources of the cost data were (e.g., expert opinion, published literature, expenditure data) and how uncertain these unit cost estimates are. If there is a lot of uncertainty in these unit cost estimates, the authors should conduct sensitivity analyses on the model input parameters to investigate the impact on the cost-effectiveness results. | We thank the reviewer for this comment and helping to highlight the importance of conveying uncertainty in model-based estimates. To the reviewers first point, there was some difficulty in securing unit costs for this study. As mentioned in the previous round of comments, we report all HIV program and modality unit costs, accompanied with program descriptors listing the source of each unit cost and references in the supplementary information. We agree with the reviewer, however, that this information could be presented in the main text. As such, we have moved this table from the supplementary information and inserted it in Table 1 of the main text. The unit costs reported in this table represent lower and upper and lower ranges.  Part of the cost function building process is a validation that cost, and coverage data fit within the understanding of the epidemic.  We also discuss the importance of recognizing that modelling studies are based on imperfect information and study findings should therefore be viewed with caution, in the limitations section of the manuscript. This has now been further emphasized in this iteration of the manuscript. We also point the reviewer to the uncertainty bounds which we include in our supplementary information. These are ranges of uncertainty estimates around results provided for projected infections and deaths (Figure S3). Our approach to this uncertainty is to randomly perturb key parameters within the model to determine the overall effect on projections. We do not think that conducting a sensitivity analysis fits in this instance. During the model calibration process, we the modeled trends to a variety of data points and estimates which include accepted estimates of HIV prevalence, for which our modeled curves include uncertainty (Figure S2). Therefore, we are as confident in our unit cost values as they were reviewed by national AIDS program representatives, along with other model parameters and assumptions customized to fit the HIV epidemic context for Eswatini. We feel that a sensitivity analysis isolated to unit costs therefore does not make sense in isolation. | We have provided descriptions of the process of acquiring each unit cost along with ranges and references in a table in the main text (previously in the supplementary material). We have emphasized the importance of recognizing results as modelled estimates in the limitations section. We have also pointed readers to our uncertainty analysis provided in the supplementary material. |
| **Minor comments** |  |  |
| The introduction seems a bit long for the introduction of an abstract. Can the authors shorten this paragraph by leaving some of the details out? For example, the age range of adults can be removed (“, with 14 infections/1000 adults in 2017.”). Remove the “over 2017.” in the next sentence. | Extraneous details have been removed from the abstract introduction, and the abstract in general. | Appropriate changes made to abstract. |
| Also, please rephrase the last sentence of the introduction so it reads as the objective of the study. The aim/objective of the study is …. | Thank you, this sentence has been revised accordingly. | This sentence now reads: To make further progress towards this target and to achieve maximum health gains, this study aims to model optimized investments of available HIV resources. |
| Methods The second sentence is too long. Please cut this sentence in (at least) 2 sentences. | This sentence has been split in two and shortened to improve clarity. | Appropriate change made. |
| Findings I would mention first what changes have to be made in the adoption/funding of HIV programs when we allocate based on cost-effectiveness. Next, mention the consequences that those changes have in the prevention of infections and deaths. | Thank you, this has been altered in the text. The descriptions of specific programs have been limited to the main text to maintain brevity in the abstract. | First sentence of results in abstract now reads: Under an optimized budget, prioritizing HIV testing for the general population followed by key preventative interventions may result in approximately… |
| Significance  Add “relatively” before “short five-year timeframe”. | Thank-you, this has been added. | Appropriate change made. |
| A budget can either be optimally spend or not optimally spend. I don’t think there can be an increase in an optimized budget. Please consider removing “increased” in this sentence. | Thank-you for the suggestion. This sentence has been poorly phrased in previous versions of the manuscript. We are describing a situation where a budget is increased, and then this new total budget is optimized. As presented in our analysis we provide several examples of this and highlight how the proportions of the total budget spent on different programs differ as total overall budget levels change. We have re-written this sentence to clarify. | Sentence now reads: ….even under a substantially increased and optimized budget level…. |
| The government is aiming for a 85% decrease in new infections and optimizing the current budget will result in a 2% reduction? There is a huge gap there. I would love to see what the authors think the implications of these findings are. Is the 85% completely unreasonable or are there additional ways to get to a higher percentage of reductions? For example, increasing the budget to what amount will approach the 85% or is 85% completely unreasonable? Mention what a reasonable increase in budget would do (and even then we would not come close to 85%). | The reviewer may have misunderstood our intention with this paragraph. The eNSF aims for an 85% reduction 2018 to 2022. Our results demonstrate an additional 2% reduction over baseline possible through optimization. This brings the total reduction to 45%. We discuss primary factors for this in the discussion section. We have removed the specific from the abstract but have clarified the total reductions in the results section. | First paragraph of the results section has been updated. |
| Introduction  Please read through one more time to improve some of the English language (e.g., 3rd paragraph: change sentence in “Reaching these HIV reduction targets in E. will be dependent on the availability of resources.” Etc.). | We thank the reviewer for their comments, this change has been made and the writing has been thoroughly reviewed and tightened throughout. | Appropriate change made and thorough review/English language editing performed |
| Methods The authors state “Drawing from a previous modelling exercise (13), the national model for Eswatini was updated in consultation with partners from the Eswatini Ministry of Health and the World Bank Group.”. Can the authors be a bit more specific about how the current study differs from the 2018 Lancet analysis (i.e., what was updated)? | We thank the reviewer for highlighting this point of clarification. We have added how this model was used to inform our current study. | The first paragraph and section of the methods section has been re-written and re-organized. |
| What do the authors mean with we initialized the model in 2000 in the sentence “We initialized the model in 2000 and produced projections from 2019 to 2023.”? Please clarify. Did you start using the model in 2000 or did you run it from 2000 until 2023 and using the 2019-2023 period for the current analysis? | The optima model provides most likely estimates of epidemic trends fitted to real data, then uses these trends to project future scenarios. We refer to initializing the model in 2000 in that we initialize our trend estimation in 2000 and run it up until 2018. Results from 2019 – 2023 are projections based on these estimates. We have clarified this in text. | The methods section has been updated appropriately. |
| Results The figures could use some footnotes to remind the reader about what the different scenarios represent. | The figures are provided with axis labels, legends, and full descriptions in text and in titles. Could the author please clarify which information they would like to see. |  |
